# Long COVID: Nueva herramienta para evaluar manifestaciones y condiciones

**DOI:** 10.15446/rsap.V26n2.116477

**Published:** 2024-03-01

**Authors:** Roxana De Las Salas

**Affiliations:** 1 Enf. M. Sc. Farmacología. Ph. D. Ciencias Farmacéuticas. Departamento de Enfermería, Universidad del Norte. Barranquilla, Colombia. rdelassalas@uninorte.edu.co Universidad del Norte Departamento de Enfermería Universidad del Norte Barranquilla Colombia rdelassalas@uninorte.edu.co

## FONDO

Según el Centro para el Control y la Prevención de Enfermedades (CDC, por su sigla en inglés), "el COVID prolongado (Long COVID, por su denominación en inglés) se define como una condición crónica que ocurre después de la infección por SARS-CoV-2 y está presente durante al menos 3 meses". Las manifestaciones y condiciones clínicas prolongadas de COVID pueden aparecer, perdurar, resolverse y reaparecer durante semanas y meses. Las manifestaciones clínicas o condiciones del COVID prolongado pueden ser de leves a graves y pueden requerir atención multidisciplinaria e integral, respaldada por herramientas de valoración específicas. Sin embargo, aún falta conciencia y herramientas disponibles para evaluar esta afección 1.

## PONIENDO LA PROPUESTA EN CONTEXTO

Los profesionales de la salud, los académicos y los investigadores necesitan herramientas válidas y confiables para evaluar la variedad de manifestaciones clínicas y condiciones que les ayuden a establecer nuevas terapias y brindar la mejor atención posible. Por eso, en la Universidad del Norte se ha desarrollado una herramienta integral que apoya la valoración de las manifestaciones clínicas del COVID prolongado para su uso en investigación y atención sanitaria. La herramienta LONG COVID-CMAT (para la valoración de manifestaciones clínicas) fue validada según evidencia científica y guías de práctica clínica sugeridas mediante la realización de un estudio de métodos mixtos. El LC-CMAT se propuso con base en una revisión sistemática de las manifestaciones clínicas de COVID prolongado. Luego, se realizó un método Delphi de consenso con profesionales de la salud y académicos de los campos de medicina, epidemiología clínica, enfermería, fisioterapia y farmacología (n = 13).

**Figura 1:**
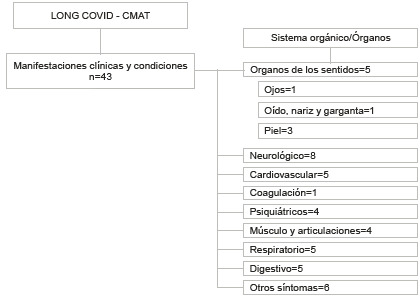
Estructura del LC-CMAT LONG COVID - CMAT

La herramienta LC-CMAT permite valorar las manifestaciones clínicas y las condiciones en adultos (≥ 18 años) con "condición post COVID-19" o "COVID prolongado". La herramienta LC-CMAT está compuesta por 43 manifestaciones y condiciones clínicas organizadas por sistemas u órganos funcionales (Figura 1). Posteriormente a la valoración, la herramienta es capaz de identificar no solo la presencia de síntomas, sino también su gravedad (leve, moderada y grave). Nuestro LC-CMAT es una herramienta validada y confiable, disponible para respaldar la valoración de COVD prolongado, y está dirigida a profesionales de la salud.

## CONCLUSIONES

Se recomienda apoyar la práctica clínica, la evaluación y la toma de decisiones utilizando esta herramienta. Al mismo tiempo, es preciso considerar el COVID prolongado como un síndrome compuesto por síntomas cognitivos, somáticos y conductuales que pueden durar varios meses o más. El COVID prolongado puede ser motivo de consulta y requerir seguimiento, tratamiento y manejo 2. Por lo tanto, este es también un llamado a todos los profesionales de la salud.
