# Supplementary material for: Long COVID: Nueva herramienta para evaluar manifestaciones y condiciones
Source: Rev Salud Publica (Bogota). 2024 Mar 1;26(2):116477. [Article in Spanish] doi: 10.15446/rsap.V26n2.116477 (PMC11665070; doi:10.15446/rsap.V26n2.116477)
Supplement: Translation

# Translation: Long covid: new tool to assess clinical manifestations and conditions

**Authors:** Roxana De Las Salas

**Affiliations:** 1 RN. M. Sc. Pharmacology. Ph. D. Pharmaceutical Sciences, Department of Nursing. Universidad del Norte. Barranquilla, Colombia. rdelassalas@uninorte.edu.co

## BACKGROUND

According to the Centers for Disease Control and Prevention (CDC), "Long COVID is defined as a chronic condition that occurs after SARS-CoV-2 infection and is pre-sent for at least 3 months". Long COVID clinical manifestations and conditions might appear, endure, resolve, and reappear during weeks and months. LONG COVID ranges could be from mild to severe and may require multidisciplinary and comprehensive care supported by specific assessment tools. However, there is a lack of awareness and available tools to assess this condition 1.

## PLACING THE PROPOSAL IN CONTEXT

Healthcare professionals, academics, and researchers need valid and reliable tools for assessing the range of symptoms and conditions to help them establish new therapies and provide the best possible care. That is why a comprehensive tool that can assess the clinical manifestations of Long COVID has been developed at the Universidad del Norte for use in research and clinical care. The LONG COVID-CMAT (Clinical manifestation assessment tool) was validated according to scientific evidence and suggested clinical practice guidelines in a mixed-methods study. The LC-CMAT was proposed based on a systematic review of Long COVID clinical manifestations. Then, a consensus Delphi method was performed with healthcare professionals and academics from the medical, epidemiology, nursing, physiotherapy, and pharmacology fields (n=13).

The LC-CMAT tool assesses clinical manifestations and conditions in adults (18+ years) with "post COVID-19 condition" or "Long COVID". The LC-CMAT is composed of 43 clinical manifestations and conditions organized by functional systems or organs (Figure 1). After the holistic assessment, the tool identifies not only the symptom's presence but severity (mild, moderate, and severe). Our LC-CMAT is a validated and feasible tool available to support Long COVD assessment led by healthcare professionals.

**Figure 1:** Structure of the LC-CMAT LONG COVID - CMAT

## CONCLUSIONS

It is recommended to support their clinical practice, assessment, and decision-making on this tool. At the same time, this is an alert made to consider Long COVID as a syndrome consisting of cognitive, somatic, and behavioral symptoms that may last several months or more. Long COVID may be the cause of clinical consultation, and require follow-up, treatment, and management 2. This is also a call for all healthcare professionals.
